# Development and internal validation of a preliminary model for *Helicobacter pylori* infection: a cross-sectional study

**DOI:** 10.3389/fmed.2026.1882621

**Published:** 2026-09-16

**Authors:** Zihao Shen, Xixi Yu, Mingcheng Liu, Yincong Luo, Xuan Li, Mingjuan Du, Piao Yuan, Xiqing Ye, Yirong Liu

**Affiliations:** 1 Clinical Medical College, Qinghai University, Xining, China; 2 Department of Otorhinolaryngology, Union Hospital, Tongji Medical College, Huazhong University of Science and Technology, Wuhan, China; 3 Department of Gastroenterology, Xining No. 1 People’s Hospital, Xining, China; 4 West China School of Medicine, Sichuan University, Chengdu, China; 5 Department of General Medicine, Xining No. 1 People’s Hospital, Xining, China

**Keywords:** calibration curves, decision curve analysis, *Helicobacter pylori*, internal validation, prediction model

## Abstract

**Background:**

Although *Helicobacter pylori* (Hp) infection is a major global public health challenge, clinically applicable tools for individualized risk stratification remain limited, particularly in geographically diverse and high-altitude populations.

**Objective:**

To develop and internally validate a clinical prediction model for Hp infection using demographic, lifestyle, altitude-related, and clinical variables.

**Methods:**

This multicenter cross-sectional study included 2034 participants recruited from three medical institutions in Qinghai Province, China, between November 2025 and March 2026. Hp infection status was determined using the ^13C-urea breath test. Participants were randomly assigned to a training set (*n* = 1423) and a validation set (*n* = 611). Candidate predictors associated with Hp infection in univariable logistic regression analysis (*P* < 0.05) were assessed for multicollinearity and subsequently entered into least absolute shrinkage and selection operator (LASSO) regression with 10-fold cross-validation. Predictors selected by LASSO were then included in a multivariable logistic regression model. A prediction model presented as a nomogram was subsequently developed. Model performance was assessed using receiver operating characteristic (ROC) analysis, calibration curves, and decision curve analysis (DCA).

**Results:**

Seven predictors were ultimately retained in the final model, including educational level, vegetable consumption, tooth brushing frequency, halitosis, family history of Hp infection, chronic gastritis, and high-altitude residence. The model demonstrated good discrimination, with area under the ROC curve (AUC) values of 0.774 in the training set and 0.756 in the validation set. Calibration analysis demonstrated good agreement between predicted and observed probabilities, with mean absolute errors of 0.018 and 0.037 in the training and validation sets, respectively. Decision curve analysis demonstrated consistent net clinical benefit in both sets.

**Conclusions:**

We developed and internally validated a prediction model for Hp infection incorporating seven readily obtainable variables. The model demonstrated satisfactory discrimination, calibration, and clinical utility and may facilitate individualized risk stratification and targeted screening strategies, particularly in high-altitude and geographically diverse populations.

## 1 Introduction

*Helicobacter pylori* (Hp) is a Gram-negative, spiral-shaped, microaerophilic bacterium that colonizes the gastric mucosa of humans. It is estimated that more than half of the global population is infected with Hp, although the prevalence varies substantially across geographic regions, ethnicity, and socioeconomic conditions (1, 2). Persistent Hp infection is a well-established risk factor for chronic gastritis, which can progress to severe gastroduodenal pathologies, including peptic ulcer, gastric cancer and gastric mucosa-associated lymphoid tissue lymphoma (3, 4). As a class I carcinogen defined by the World Health Organization, Hp is responsible for the majority of non-cardia gastric cancers worldwide, and approximately 850,000 cancer cases annually are attributable to this infection (5). Given the substantial global burden of Hp-related diseases, early identification of high-risk individuals is of considerable importance for targeted screening, prevention, and eradication strategies.

Accumulating evidence suggests that Hp infection is influenced not only by host susceptibility but also by complex interactions among environmental, behavioral, and socioeconomic factors (6). Previous studies have identified multiple factors associated with infection, including lower educational attainment, poor sanitation and hygiene practices, crowded living conditions, family clustering, dietary habits, and oral health status (7). Geographic and environmental factors may also play important roles in shaping regional differences in infection prevalence (8). In western China, particularly in high-altitude areas such as the Qinghai-Tibet Plateau, populations are exposed to unique environmental conditions, ethnic lifestyles, and dietary patterns (9, 10). These region-specific characteristics may influence Hp transmission and persistence through pathways related to sanitation, nutrition, oral-gastric microbial ecology, and healthcare accessibility (11). However, these factors remain insufficiently integrated into current risk assessment approaches.

In recent years, increasing attention has been directed toward population-based “screen-and-treat” strategies for Hp infection, particularly in regions with a high burden of gastric cancer (12). Although non-invasive diagnostic tests for Hp infection are widely available, universal population-based testing may not be practical in geographically remote or resource-limited settings because of limited healthcare accessibility and resource constraints. In this context, risk-stratification tools may serve as an initial assessment approach to identify individuals who are more likely to benefit from further Hp testing and to improve the efficiency of screening strategies. Most existing studies have primarily focused on individual variables associated with Hp infection or epidemiological associations (13, 14), whereas clinically applicable prediction models remain limited. Furthermore, several previously reported prediction models were developed in geographically restricted or relatively homogeneous populations, which may limit their generalizability to geographically diverse settings (15). Importantly, prediction studies incorporating high-altitude environmental exposure and region-specific lifestyle factors remain scarce, despite the substantial heterogeneity in Hp prevalence observed across western China (8, 16). Therefore, there is a need for a clinically applicable prediction model that integrates demographic, lifestyle, environmental, and medical variables to support initial risk assessment and identify individuals who may benefit from further Hp testing. Rather than identifying causal determinants, such a model is intended to integrate clinically relevant predictors to estimate an individual's probability of Hp infection.

Therefore, in the present study, we developed and internally validated a multivariable clinical prediction model for Hp infection using data from multiple regions of Qinghai Province, including both high-altitude and non-high-altitude populations. Using least absolute shrinkage and selection operator (LASSO) regression and multivariable logistic regression analysis, we developed a clinically applicable prediction model presented as a nomogram incorporating readily obtainable demographic, lifestyle, environmental, and clinical variables. The discrimination, calibration, and clinical utility of the model were comprehensively evaluated using receiver operating characteristic analysis, calibration curves, and decision curve analysis. By integrating high-altitude environmental exposure and region-specific lifestyle characteristics into predictive modeling, this study aimed to provide a practical tool for initial risk assessment to identify individuals who may benefit from further Hp testing and support precision prevention strategies for Hp infection.

## 2 Methods

### 2.1 Study design and participants

This cross-sectional study was conducted in Qinghai Province, China, using data collected between November 2025 and March 2026. Participants were consecutively recruited from routine health examination clinics and outpatient services at three healthcare institutions representing both high-altitude and non-high-altitude regions. A total of 2034 participants were included in the final analysis, including 1226 participants from Xining No.1 People's Hospital, Xining (altitude approximately 1500–2500 m above sea level), 405 participants from Yunjiakou Township Health Center, Chengdong District, Xining (altitude approximately 1500–2500 m above sea level), and 403 participants from Nangqian County, Yushu Tibetan Autonomous Prefecture (altitude approximately 3500–4500 m above sea level).

Individuals aged 3–90 years who attended the participating institutions during the study period were screened for eligibility. Participants with a previous history of Hp eradication therapy, incomplete clinical data, duplicate records, or recent use of antibiotics, proton pump inhibitors, or bismuth-containing medications within 4 weeks before ^13C-urea breath testing were excluded. Eligible participants subsequently completed the questionnaire assessment under the guidance of trained study staff. For participants who were unable to complete the questionnaire independently, the questionnaire was completed with assistance from their parents or legal guardians. All enrolled participants then underwent ^13C-urea breath testing for Hp detection. Because eligibility was confirmed before questionnaire administration, all enrolled participants completed the questionnaire, and no missing data were present for the candidate predictors included in the final analysis.

This study was approved by the Ethics Committee of Xining No.1 People's Hospital (Approval No. 2025-LLPJ-007) and was conducted in accordance with the principles of the Declaration of Helsinki. Written informed consent was obtained from all participants prior to participation. For participants younger than 18 years, written informed consent was obtained from their legal guardians.

### 2.2 Data collection and variable definition

Demographic characteristics, lifestyle factors, dietary variables, oral hygiene behaviors, medical history, and family history were collected using structured questionnaires. For pediatric participants, questionnaires were completed by parents or legal guardians when necessary. BMI was calculated as weight in kilograms divided by height in meters squared (kg/m^2^). Educational level was categorized as junior high school or below, high school, college diploma, and bachelor's degree or above. Dietary intake variables were categorized according to weekly consumption frequency as never, occasionally (<3 times/week), or frequently (≥3 times/week). Smoking status and alcohol consumption were categorized as never, former, or current. Physical activity and weekly working hours were assessed as weekly exercise duration and average working hours per week, respectively. Tooth brushing frequency was categorized as ≤1 time/day or ≥2 times/day. Sleep quality was categorized as good, fair, or poor. Halitosis was assessed by participants’ self-report based on whether they perceived themselves as having oral malodour (yes/no). Family history of Hp infection was defined as a self-reported history of a cohabiting first-degree relative who had previously been diagnosed with Hp infection. Previous Hp infection, chronic gastritis, and peptic ulcer history was obtained from questionnaire data. Participants recruited from Nangqian County were classified as high-altitude residents, whereas those recruited from the two institutions in Xining were classified as non-high-altitude residents. Because individual residential altitude was not recorded and altitude classification corresponded directly to recruitment region, high-altitude residence was treated as a region-level proxy variable rather than a direct measure of individual altitude exposure.

### 2.3 Assessment of Hp infection

Hp infection status was determined using the 13C-urea breath test diagnostic kit (Beijing Boran Pharmaceutical Co., Ltd., China). Participants were required to fast overnight or for at least 2 hours prior to testing. Two gas bags labeled with adhesive tags were provided, on which each subject completed the required study information. A baseline breath sample (0-minute sample) was collected by having subjects exhale normally into a gas bag until it was fully inflated, followed by immediate sealing of the bag. Subjects then dissolved the urea [^13^C] granules in 80–100 mL of cool potable water, consumed the solution after thorough mixing, and remained seated at rest throughout the test period. Using the same collection procedure, a second breath sample was obtained 30 minutes after administration of the labeled urea. Both the 0-minute and 30-minute breath samples were subsequently analyzed for ^13^CO₂ content using the dedicated instrument. The test results were expressed as the per mil difference value (δ‰), calculated as: δ‰ = [(^13^C-isotope abundance of the test sample−^13^C-isotope abundance of the reference sample)/^13^C-isotope abundance of the reference sample] × 1000. For the diagnosis of Hp infection, the final detection value was determined by subtracting the δ‰ value of the 0-minute baseline sample from that of the 30-minute post-administration sample (δ‰(30 min)−δ‰(0 min)), with a detection value of ≥ 4.0 ± 0.4 defined as the cut-off for a positive Hp result.

### 2.4 Statistical analysis

The dataset was randomly divided into a training set and a validation set at a ratio of 7:3 using simple random sampling implemented in R with a fixed random seed (set.seed = 123). The training cohort was used for model development and predictor selection, whereas the validation cohort was used for internal validation of model performance. Characteristics of participants between the training and validation sets were compared using the Mann–Whitney U test for continuous variables and the chi-square test or Fisher's exact test for categorical variables, as appropriate. Univariable logistic regression analysis was first performed in the training cohort to evaluate the crude association between each candidate predictor and Hp infection. Predictors with a univariable *P* value <0.05 were retained as eligible predictors for subsequent feature selection. This preliminary screening step was used as a pragmatic approach to reduce the broad set of questionnaire-derived variables and improve the parsimony and interpretability of the prediction model. Multicollinearity was then assessed among the screening-eligible predictors using the variance inflation factor (VIF). A total of 35 predictors met the univariable screening criterion, and none was excluded on the basis of the VIF assessment because no evidence of severe multicollinearity was identified. Consequently, all 35 predictors were entered into the least absolute shrinkage and selection operator (LASSO) regression model for further feature selection. Categorical variables were represented as dummy variables, and continuous predictors were automatically standardized before model fitting. LASSO regression analysis with 10-fold cross-validation was conducted to reduce dimensionality and prevent model overfitting. The optimal penalty parameter was selected according to the 1-standard error criterion (lambda.1se) to obtain a more parsimonious model. Predictors with nonzero coefficients identified by LASSO regression were subsequently entered into a multivariable logistic regression model to construct the final prediction model. Regression coefficients, odds ratios (ORs), 95% confidence intervals (CIs), and *P* values were calculated. A nomogram was established based on the final multivariable logistic regression model to provide individualized risk estimation for Hp infection. Model discrimination was evaluated using receiver operating characteristic (ROC) curves and the area under the ROC curve (AUC) in both the training and validation sets. The optimal cutoff value was determined using the Youden index (sensitivity + specificity−1), with the cutoff corresponding to the maximum Youden index considered optimal. Model calibration was assessed using calibration curves generated by bootstrap resampling with 1000 repetitions. Clinical utility of the prediction model was evaluated using decision curve analysis (DCA). All statistical analyses were performed using R software (version 4.1.1; R Foundation for Statistical Computing, Vienna, Austria). A two-sided *P* value <0.05 was considered statistically significant.

## 3 Results

### 3.1 Characteristics of participants in the training and validation sets

A total of 2034 participants were included in this study, comprising 1423 participants in the training set and 611 participants in the validation set. The median age was 39 years in both sets, and the prevalence of Hp infection was comparable between the training and validation sets (52.64% vs. 51.72%). Baseline characteristics of the participants are presented in Supplementary Table S1. No substantial differences were observed between the two sets for most demographic and clinical variables. Significant differences were identified in educational level (*P* = 0.020).

### 3.2 Univariable analysis of candidate predictors for Hp infection

Univariable logistic regression identified 35 predictors that met the screening criterion of *P* < 0.05. Key results are summarized in Table 1, and detailed results, including VIF values, are presented in Supplementary Table S2. Notable predictors included age, ethnicity, educational level, high-altitude residence, dietary factors (butter tea, vegetable, raw food, and fruit consumption), hygiene-related behaviors (tooth brushing frequency and hand hygiene before meals and after toileting), halitosis, family history of Hp infection, and chronic gastritis. No evidence of severe multicollinearity was identified among the 35 screening-eligible predictors (all VIFs < 10); therefore, all 35 predictors were entered into the LASSO regression model for further feature selection.

**Table 1:** Univariable analysis of candidate predictors for Hp infection.

Variable	OR (95% CI)	*P* value
Age, years	0.992 (0.986–0.998)	0.007
Ethnicity (Tibetan vs. Han ethnicity)	5.263 (3.912–7.080)	<0.001
Educational level (per category increase)	0.625 (0.572–0.682)	<0.001
High-altitude residence (yes vs. no)	8.011 (5.604–11.453)	<0.001
Butter tea consumption (per category increase)	2.065 (1.689–2.525)	<0.001
Raw food consumption (per category increase)	1.540 (1.255–1.891)	<0.001
Fruit consumption (per category increase)	0.644 (0.527–0.788)	<0.001
Vegetable consumption (per category increase)	0.495 (0.400–0.611)	<0.001
Tooth brushing frequency (≥2 vs. ≤ 1 times/day)	0.400 (0.322–0.498)	<0.001
Hand hygiene before meals and after toileting (yes vs. no)	0.350 (0.247–0.498)	<0.001
Halitosis (yes vs. no)	2.432 (1.918–3.084)	<0.001
Family history of Hp infection (yes vs. no)	1.729 (1.365–2.191)	<0.001
Chronic gastritis (yes vs. no)	2.196 (1.691–2.852)	<0.001

Odds ratios (ORs) and 95% confidence intervals (CIs) were estimated using univariable logistic regression analysis of candidate predictors. The reference categories for binary variables are indicated in parentheses.

Hp, *Helicobacter pylori*.

### 3.3 Feature selection using LASSO regression model

LASSO regression with 10-fold cross-validation was performed for feature selection (Figure 1). Using the lambda.1se criterion (*λ* = 0.032), seven variables with non-zero coefficients were identified and retained for subsequent multivariable logistic regression analysis. These variables included educational level, vegetable consumption, frequency of tooth brushing, halitosis, family history of Hp infection, chronic gastritis, and high-altitude residence.

**Figure 1:**
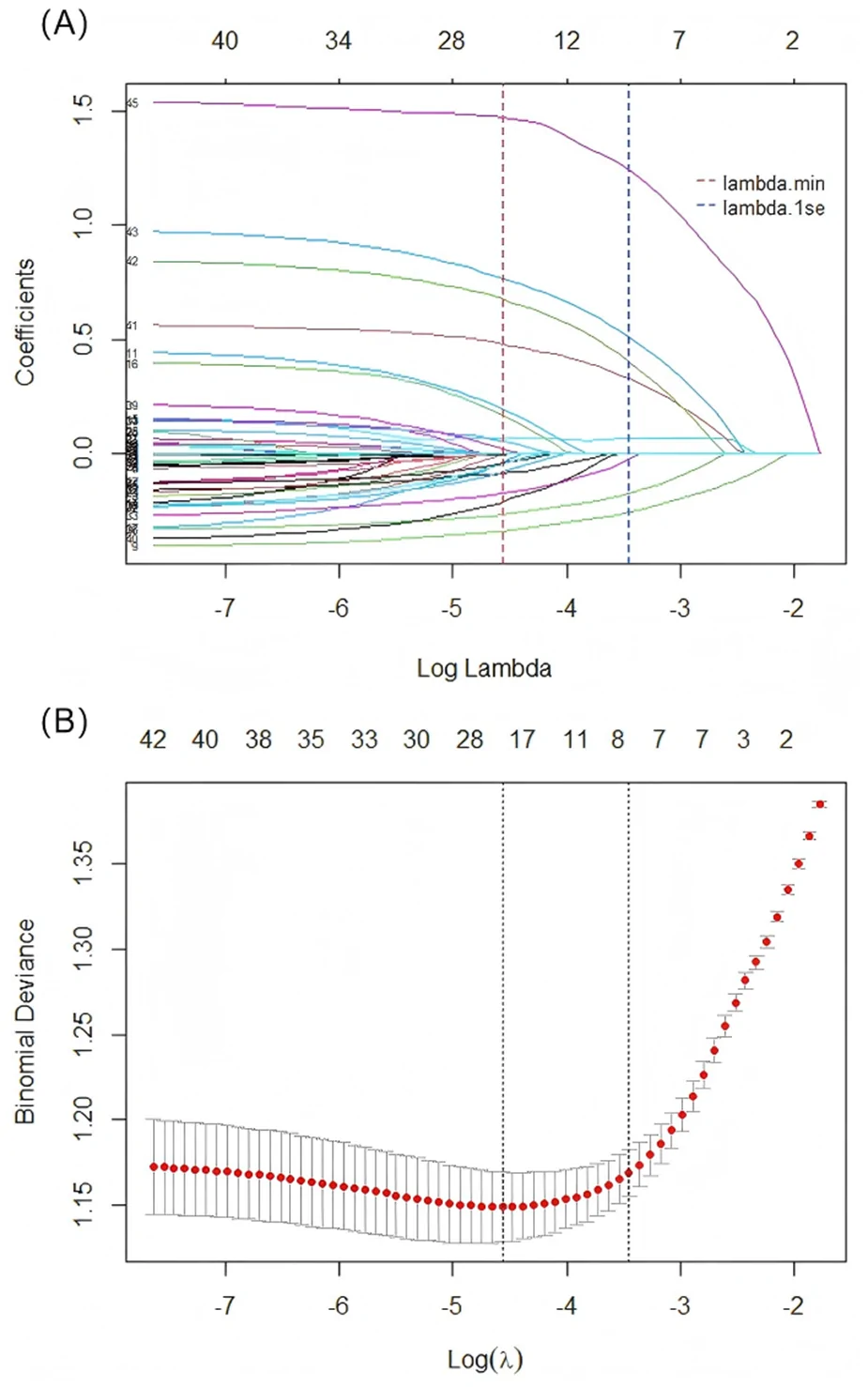
Feature selection using the least absolute shrinkage and selection operator (LASSO) regression model. **(A)** LASSO coefficient profiles of the 35 candidate predictors. Each colored line represents the coefficient trajectory of an individual predictor across different values of the penalty parameter *λ*. **(B)** Ten-fold cross-validation plot for tuning parameter selection in the LASSO model. The dotted vertical lines indicate the optimal lambda values based on the minimum criteria (lambda.min) and the 1-standard error criteria (lambda.1se). The lambda.1se criterion was selected to obtain a more parsimonious model, resulting in seven predictors with nonzero coefficients for subsequent multivariable logistic regression analysis.

### 3.4 Development of the prediction model for Hp infection

A multivariable logistic regression model was constructed using the seven variables selected by LASSO regression (Table 2). Higher educational level, more vegetable consumption and more frequent tooth brushing were associated with lower odds of Hp infection, whereas halitosis, family history of Hp infection, chronic gastritis, and high-altitude residence were associated with increased odds of infection. A nomogram was constructed based on the final multivariable logistic regression model to estimate the individualized probability of Hp infection (Figure 2). For individual risk assessment, each predictor is assigned a corresponding point value, and the points are summed to obtain a total score corresponding to the predicted probability of Hp infection. Individuals with multiple risk factors therefore receive higher total scores and higher predicted risks.

**Table 2:** Multivariable logistic regression model for predicting Hp infection.

Variable	*β*	OR (95% CI)	*P* value
Educational level (per category increase)	−0.382	0.683 (0.616–0.755)	<0.001
Vegetable consumption (per category increase)	−0.286	0.751 (0.583–0.968)	0.027
Frequency of tooth brushing (≥2 vs. ≤ 1 times/day)	−0.327	0.721 (0.560–0.929)	0.011
Halitosis (yes vs. no)	0.579	1.784 (1.362–2.342)	<0.001
Family history of Hp infection (yes vs. no)	0.834	2.303 (1.754–3.034)	<0.001
Chronic gastritis (yes vs. no)	0.901	2.462 (1.843–3.303)	<0.001
High-altitude residence (yes vs. no)	1.673	5.327 (3.589–8.063)	<0.001

*β* indicates the regression coefficient. Odds ratios (ORs) and 95% confidence intervals (CIs) were estimated using multivariable logistic regression analysis. ORs for ordinal variables represent the change in odds associated with a one-category increase.

Hp, *Helicobacter pylori*.

**Figure 2:**
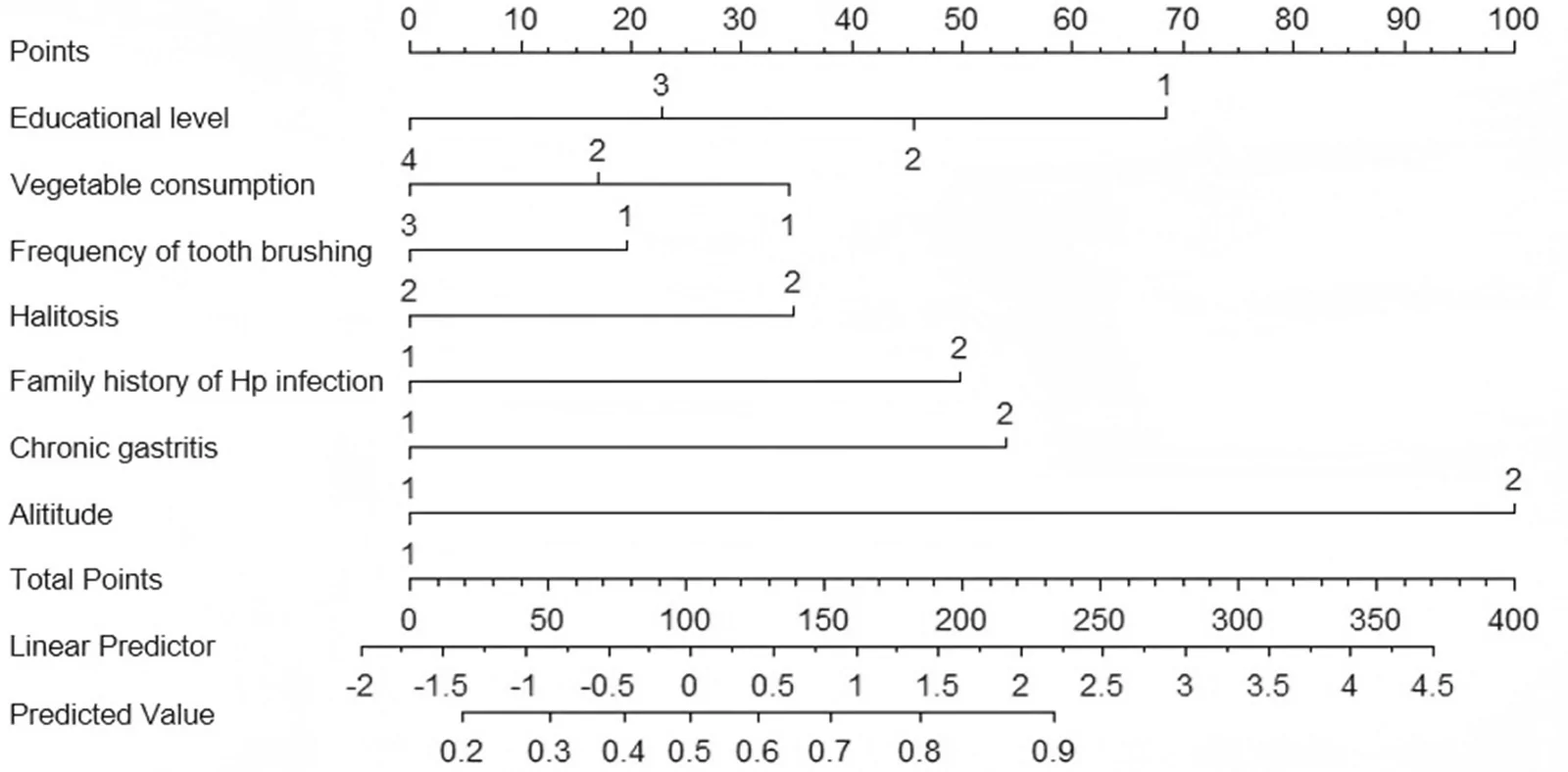
Nomogram for individualized prediction of Hp infection. For each predictor, points were assigned according to the corresponding value on the upper “Points” scale, and the total points were used to estimate the predicted probability of Hp infection. Educational level was coded as: 1 = Junior high school or below, 2 = High school, 3 = College diploma, and 4 = Bachelor's degree or above. Vegetable consumption was coded as: 1 = Never, 2 = Occasionally (<3 times/week), and 3 = Frequently (≥3 times/week). Tooth brushing frequency was coded as: 1 = ≥2 times/day and 2 = ≤1 time/day. Halitosis, family history of Hp infection, chronic gastritis, and high-altitude residence were coded as: 1 = No and 2 = Yes.

### 3.5 Discriminative ability of the prediction model

The prediction model demonstrated good discriminative performance in both the training and validation sets (Figure 3). The AUCs were 0.774 in the training set and 0.756 in the validation set. The optimal cutoff values were determined based on the Youden index. In the training set, the optimal cutoff was 0.509, corresponding to a sensitivity of 66.2%, specificity of 73.4%, and accuracy of 69.6% (Figure 3A). In the validation set, the optimal cutoff was 0.551, with a sensitivity of 60.4%, specificity of 75.3%, and accuracy of 67.6% (Figure 3B). When the cutoff derived from the training set (0.509) was applied to the validation set, the sensitivity, specificity, and accuracy were 64.2%, 69.5%, and 66.8%, respectively.

**Figure 3:**
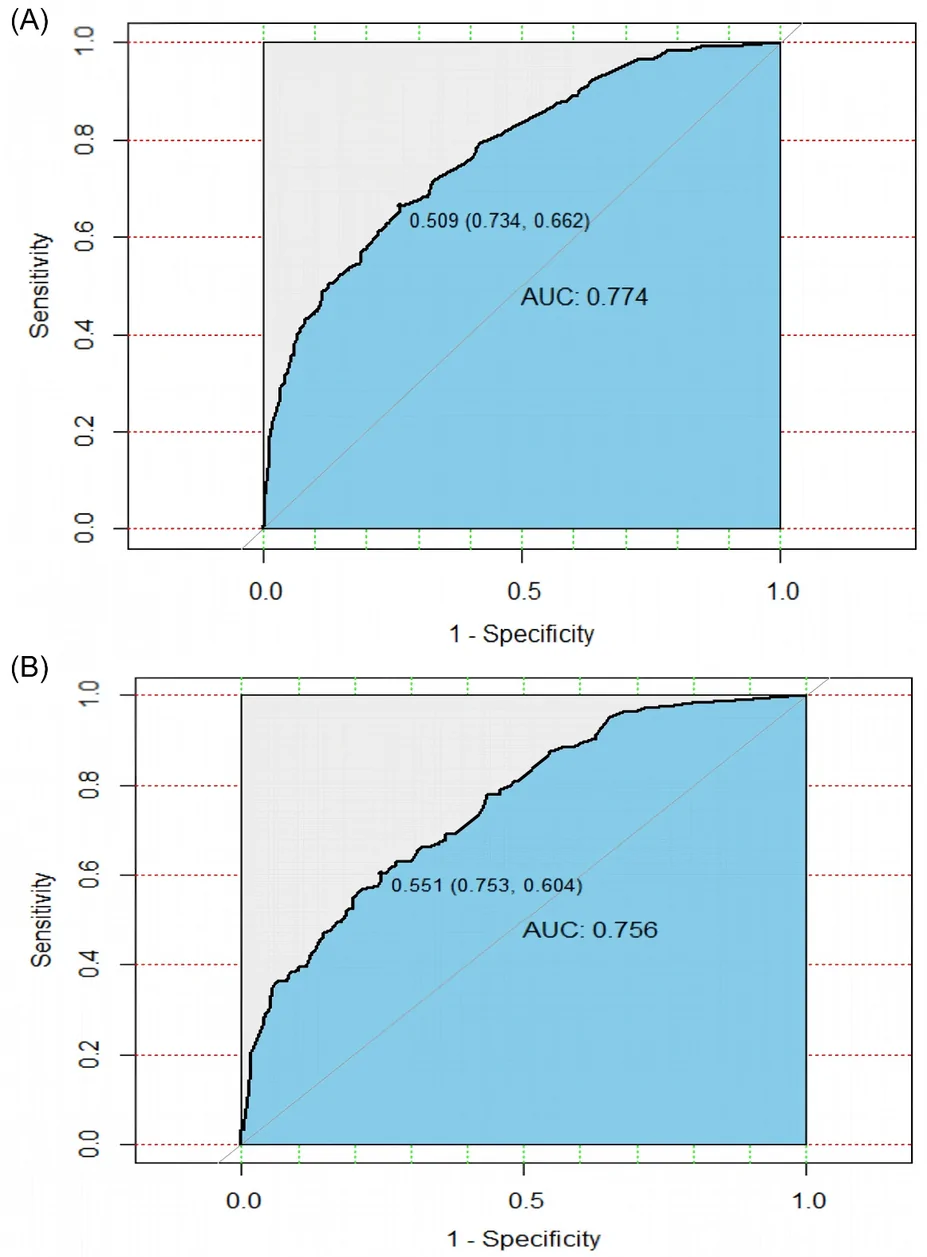
Receiver operating characteristic (ROC) curves of the final prediction model in the training and validation sets **(A)** ROC curve of the prediction model in the training set, with an AUC of 0.774. **(B)** ROC curve of the prediction model in the validation set, with an AUC of 0.756. AUC, area under the curve.

### 3.6 Calibration of the prediction model

Calibration curves of the prediction model in the training and validation sets are presented in Figure 4. In the training set, the apparent calibration curve and the bootstrap bias-corrected calibration curve were both close to the ideal reference line. The mean absolute error (MAE) was 0.018 after 1000 bootstrap resamples. In the validation set, the calibration curve also demonstrated good consistency between predicted and observed probabilities, with an MAE of 0.037. The calibration intercepts and slopes were approximately 0 and 1, respectively, in both the training and validation sets.

**Figure 4:**
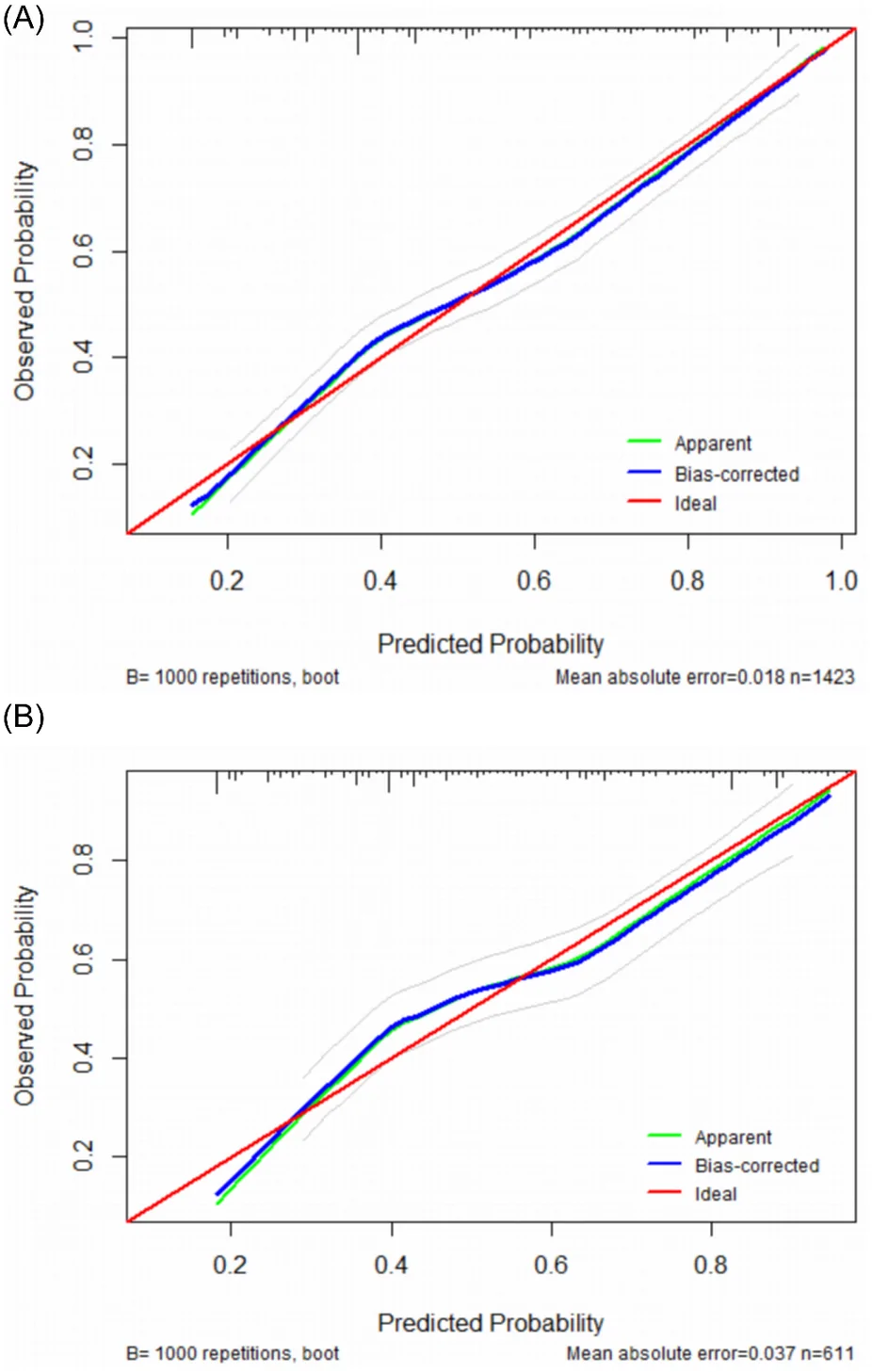
Calibration curves of the prediction model in the **(A)** training and **(B)** validation sets **(A)** calibration curve of the prediction model in the training set. **(B)** Calibration curve of the prediction model in the validation set. The red line represents the ideal calibration, the blue line represents the bias-corrected calibration estimated by bootstrap resampling, and the green line represents the apparent calibration.

### 3.7 Clinical utility of the prediction model

Decision curve analysis was performed in the training (Figure 5A) and validation sets (Figure 5B) to assess the clinical utility of the prediction model. The prediction model demonstrated a higher net benefit than the “treat-none” strategy across a wide range of clinically relevant threshold probabilities in both datasets. Compared with the “treat-all” strategy, the prediction model also showed superior net benefit at threshold probabilities below approximately 0.55. These findings indicate potential clinical utility of the model for initial risk assessment of Hp infection.

**Figure 5:**
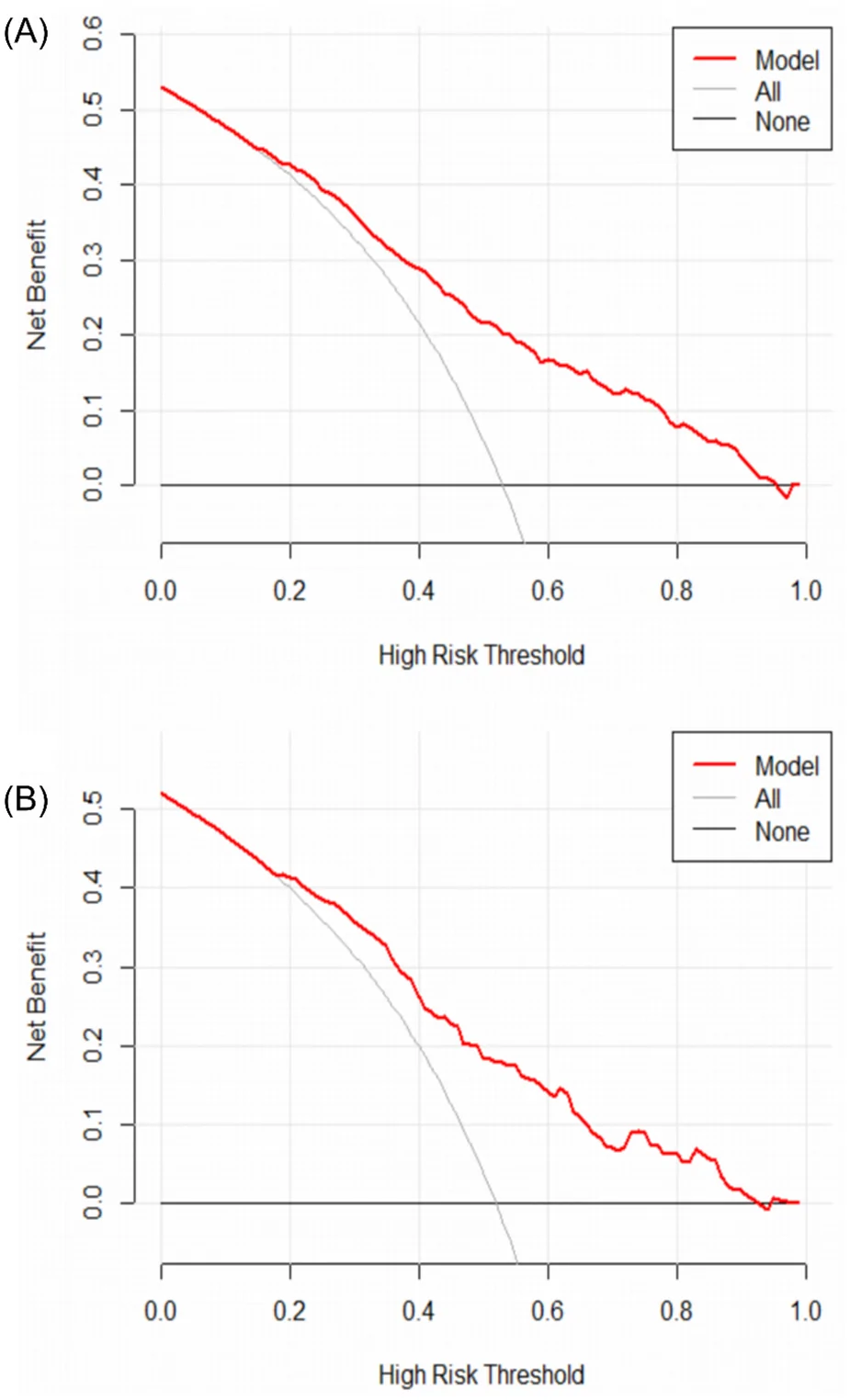
Decision curve analysis (DCA) of the prediction model in the **(A)** training and **(B)** validation sets **(A)** DCA curve of the prediction model in the training set. **(B)** DCA curve of the prediction model in the validation set. The red line represents the prediction model, the gray line represents the strategy of treating all individuals as having Hp infection, and the black line represents the strategy of treating none.

## 4 Discussion

In this study, we developed and internally validated a clinical prediction model for Hp infection using seven readily obtainable variables derived from geographically diverse populations in Qinghai Province. The final model incorporated educational level, vegetable consumption, oral hygiene–related factors, family history of Hp infection, chronic gastritis, and high-altitude residence, highlighting the combined contributions of environmental exposure, dietary patterns, oral health behaviors, and clinical history to Hp infection risk. Importantly, the model demonstrated acceptable discrimination together with satisfactory calibration and decision curve performance in both the training and validation sets, supporting its potential use as an initial risk assessment tool rather than a standalone diagnostic model. Given that all predictors are readily obtainable through routine history taking without additional laboratory testing, the proposed model may serve as an initial risk assessment tool to prioritize individuals who are more likely to benefit from confirmatory Hp testing, particularly in high-altitude and resource-limited regions where universal screening may not be feasible. Rather than replacing established diagnostic tests, the model is intended to assist in identifying individuals for whom further testing may be most appropriate. Nevertheless, its cost-effectiveness and feasibility for individual-level implementation require prospective evaluation.

Several predictors identified in the present study are consistent with previous epidemiological findings. Educational level was inversely associated with Hp infection risk, in agreement with prior studies demonstrating that lower educational status and socioeconomic are strongly associated with increased Hp prevalence (1, 17). Lower educational attainment may reflect disparities in health literacy, sanitation practices, household crowding, and healthcare accessibility, all of which may facilitate intrafamilial and environmental transmission of Hp (18, 19). Educational attainment may also reflect early-life socioeconomic conditions, which are known to influence childhood acquisition and long-term persistence of Hp infection (20–22). Similarly, family history of Hp infection remained an independent predictor in the final model, supporting previous evidence that close household contact and familial aggregation play important roles in Hp transmission dynamics (23, 24).

Oral hygiene–related variables also emerged as important predictors in our model. More frequent tooth brushing was associated with reduced odds of Hp infection, whereas halitosis was associated with increased infection risk. These findings are consistent with growing evidence suggesting that the oral cavity may serve as a potential extragastric reservoir for Hp, particularly within dental plaque, thereby contributing to bacterial persistence and gastric reinfection following eradication therapy (25, 26). Poor oral hygiene may facilitate this process by promoting dental plaque accumulation and the persistence of Hp in the oral cavity. In addition, oral microbial dysbiosis has been proposed as a potential contributor to Hp colonization, although the underlying mechanisms remain incompletely understood (27). Previous studies have also demonstrated associations between periodontal disease, oral malodor, and Hp positivity, suggesting that oral health management may represent an important adjunctive strategy for Hp prevention and eradication (28). These observations may partially explain why poor oral hygiene has been associated with persistence or recurrence of Hp infection after eradication therapy (28, 29).

One notable finding of the present study was the significant contribution of high-altitude residence and vegetable consumption to Hp infection risk. Qinghai Province is characterized by substantial geographic and environmental heterogeneity, including marked altitude differences and unique dietary patterns. High-altitude populations often experience distinct lifestyle, sanitation, healthcare accessibility, and nutritional conditions compared with non-high-altitude populations (30, 31). In addition to environmental and behavioral differences, chronic hypoxic exposure at high altitude may influence gastric mucosal physiology, local immune responses, and microbial ecology (32–34), potentially creating a microenvironment more favorable for Hp colonization and persistence. Higher meat intake and lower vegetable and fruit consumption are characteristic features of the traditional diet in high-altitude regions (35). Within this distinctive environmental and dietary context, vegetable consumption emerged as another important predictor of Hp infection. We found that greater vegetable consumption was associated with lower odds of Hp infection. Previous studies have reported that the average vegetable consumption among plateau residents are substantially below dietary recommendations and decreases with increasing altitude (36, 37). The harsh climate, short growing season, limited transportation, and traditional dietary practices jointly restrict access to and consumption of fresh vegetables in high-altitude areas. The “grains-vegetables” dietary pattern is associated with a lower prevalence of Hp infection (38). Vitamin C and dietary fiber in vegetables may help alleviate oxidative stress in the gastric mucosa, suppress inflammatory responses, preserve mucosal barrier integrity, and modulate the gastrointestinal microbiota (39, 40). However, the present study examined only the association between vegetable consumption and Hp infection. Therefore, the observed relationship may also reflect the combined influence of region-specific dietary and environmental characteristics. Further mechanistic studies are warranted to clarify the biological basis of this association.

Interestingly, ethnicity itself was not retained in the final model after LASSO selection, whereas altitude remained significant predictors. In Qinghai Province, ethnicity, altitude, and traditional dietary patterns are inherently correlated because they reflect overlapping geographic and sociocultural characteristics. To further evaluate whether this relationship introduced multicollinearity, we performed variance inflation factor (VIF) analysis. All VIF values were below the commonly accepted threshold for severe multicollinearity (all VIFs < 10), suggesting that the exclusion of ethnicity during LASSO feature selection was more likely attributable to overlapping predictive information shared with other correlated variables rather than problematic multicollinearity. Nevertheless, all high-altitude participants were recruited from Nangqian County, whereas all non-high-altitude participants were recruited from the two institutions in Xining; therefore, altitude classification was structurally aligned with recruitment site. This design prevented the independent effects of altitude and recruitment site from being disentangled, and the VIF results do not exclude potential confounding by site-level differences. These findings suggest that environmental and lifestyle-related factors may explain a substantial proportion of the observed ethnic disparities in Hp prevalence, highlighting the importance of considering region-specific exposures rather than ethnicity alone. Taken together, these findings indicate that Hp infection in high-altitude regions is unlikely to be driven by a single factor, but rather by complex interactions among environmental exposure, dietary patterns, hygiene behaviors, and household transmission dynamics. Because the high-altitude population in the present study was recruited from a single geographic region, the effects of altitude cannot be completely disentangled from coexisting ethnic, cultural, dietary, and socioeconomic characteristics. Both high-altitude residence and vegetable consumption are more appropriately regarded as composite predictors reflecting broader environmental and sociocultural exposures than as direct causal determinants of Hp infection.

Chronic gastritis was also independently associated with Hp infection in the final model. Persistent Hp colonization is a well-established cause of chronic gastric inflammation (41, 42), which explains the close association observed between these two conditions. In addition, alterations in the gastric mucosal environment associated with chronic gastritis have been proposed to contribute to the persistence of Hp, although the underlying mechanisms remain incompletely understood (43). Although chronic gastritis is widely recognized as a clinical consequence of persistent Hp infection, prediction models are designed to optimize predictive performance rather than establish causal relationships. Therefore, chronic gastritis was included as a clinically relevant predictor because its presence provides useful information for estimating the probability of current Hp infection, rather than implying that it is a causal risk factor.

Although plausible biological and epidemiological explanations exist for several predictors included in the model, it should be emphasized that the present prediction model was developed for risk stratification rather than causal inference. Some predictors, particularly vegetable consumption and high-altitude residence, are likely to represent surrogate markers of broader environmental, dietary, and sociocultural exposures rather than direct determinants of Hp infection. Likewise, variables such as educational level and halitosis may reflect underlying socioeconomic conditions or oral health status associated with infection risk. Therefore, the predictive value of these variables does not necessarily imply a direct mechanistic relationship with Hp colonization. Further mechanistic and longitudinal studies are warranted to clarify the biological pathways underlying these observed associations.

Building upon previous epidemiological studies investigating risk factors associated with Hp infection (44, 45), the present study further explored individualized risk prediction and clinical applicability using an integrated multivariable modeling approach. We incorporated a relatively comprehensive set of demographic, environmental, dietary, oral hygiene, and medical variables from both high-altitude and non-high-altitude populations. Moreover, the use of LASSO regression enabled dimensionality reduction and construction of a relatively parsimonious model while minimizing the risk of overfitting. The comparable predictive performance observed in the training and validation cohorts, together with favorable calibration and decision curve analysis results, further supports the robustness of the model within the present study population and its potential utility as an initial risk assessment tool.

The proposed prediction model may have several practical implications. First, it provides a simple and noninvasive pre-screening tool for identifying individuals who may benefit from confirmatory Hp testing using routinely obtainable clinical and lifestyle information. Second, rather than replacing established diagnostic methods, the model may complement existing screening strategies by helping prioritize individuals for subsequent diagnostic testing, particularly in resource-limited settings. Third, several predictors included in the model represent modifiable behavioral characteristics, suggesting potential opportunities for population-level health education and preventive interventions. Given the moderate discriminative performance, the model should be considered an adjunctive tool for preliminary risk assessment rather than a substitute for established diagnostic methods.

Several limitations should be acknowledged. First, this was a cross-sectional study, and causal relationships between predictors and Hp infection could not be established. Second, the use of univariable *P*-value screening before LASSO may have excluded predictors with limited marginal associations but potential predictive value when considered jointly with other predictors and may also have increased the instability of predictor selection. In addition, although internal validation demonstrated satisfactory model performance, the robustness of the selected predictor set should be further assessed in analyses incorporating the complete set of candidate predictors, and external validation in independent populations and other geographic regions remains necessary before widespread clinical application. Third, high-altitude residence was defined according to recruitment region rather than individual residential altitude, and all high-altitude participants were recruited from a single geographic area. Because altitude classification was structurally aligned with recruitment site, the predictive contribution of high-altitude residence could not be separated from site-related differences in ethnicity, cultural and dietary patterns, socioeconomic conditions, population composition, and healthcare accessibility. Site-based validation of the original model was not feasible because excluding the only high-altitude site would have eliminated variation in the high-altitude predictor from the development data. Future validation should therefore include multiple independent recruitment sites at both high and non-high altitudes. Fourth, the study population was recruited exclusively from Qinghai Province through routine health examination clinics and outpatient services. Consequently, participants may have had relatively higher educational attainment and health awareness than the general population, which may have introduced selection bias and limited the generalizability of the model to populations with different demographic characteristics and healthcare utilization patterns. Finally, several variables, including dietary habits, oral hygiene behaviors, and halitosis status, and family history of Hp infection, were self-reported and therefore subject to potential recall and reporting bias. In particular, halitosis was assessed based on self-perceived oral malodour rather than objective clinical measurements, and family history of Hp infection was based on participants’ reports of previously diagnosed infection among cohabiting first-degree relatives. These factors may have resulted in some degree of misclassification and affected the accuracy of the observed associations.

## 5 Conclusion

In this multicenter cross-sectional study conducted in Qinghai Province, we developed and internally validated a prediction model for Hp infection incorporating educational level, vegetable consumption, oral hygiene behaviors, family history of Hp infection, chronic gastritis, and high-altitude residence. The findings suggest that Hp infection risk is shaped by the combined influence of environmental exposure, dietary patterns, oral health behaviors, and clinical history, particularly in geographically and ethnically diverse populations. The proposed model may serve as a practical tool for identifying individuals at increased risk of Hp infection and may assist in optimizing targeted screening and prevention strategies in resource-limited and high-altitude regions.

## Data Availability

The raw data supporting the conclusions of this article will be made available by the authors, without undue reservation.
