# Supplementary material for: Development and internal validation of a preliminary model for Helicobacter pylori infection: a cross-sectional study
Source: Front Med (Lausanne). 2026 Sep 16;13:1882621. doi: 10.3389/fmed.2026.1882621 (PMC13623725; doi:10.3389/fmed.2026.1882621)
Supplement: Supplementary file 1 [file Table1.docx]

**Table S1. Characteristics of participants in the training and validation sets**

| **Variable** | **Training set (n = 1423)** | **Validation set (n = 611)** | ***P* value** |
| --- | --- | --- | --- |
| Age, years | 39.00 (27.00, 55.00) | 39.00 (26.00, 53.50) | 0.885 |
| Sex, n (%) |  |  | 0.324 |
| Male | 597 (41.95) | 242 (39.61) |  |
| Female | 826 (58.05) | 369 (60.39) |  |
| Ethnicity, n (%) |  |  | 0.089 |
| Han | 839 (58.96) | 372 (60.88) |  |
| Hui | 178 (12.51) | 61 (9.98) |  |
| Tibetan | 350 (24.60) | 142 (23.24) |  |
| Other | 56 (3.94) | 36 (5.89) |  |
| BMI, kg/m² | 22.92 (20.40, 25.39) | 22.64 (20.01, 25.32) | 0.309 |
| Education level, n (%) |  |  | 0.020 |
| Junior high school or below | 681 (47.86) | 266 (43.54) |  |
| High school | 198 (13.91) | 71 (11.62) |  |
| College diploma | 191 (13.42) | 84 (13.75) |  |
| Bachelor’s degree or above | 353 (24.81) | 190 (31.10) |  |
| High-altitude residence, n (%) |  |  | 0.238 |
| No | 1137 (79.90) | 502 (82.16) |  |
| Yes | 286 (20.10) | 109 (17.84) |  |
| Weekly working hours | 35.00 (5.00, 40.00) | 36.00 (9.00, 40.00) | 0.134 |
| Physical activity, h/week | 5.00 (3.00, 8.00) | 4.00 (2.00, 8.00) | 0.117 |
| Sleep quality, n (%) |  |  | 0.332 |
| Good | 575 (40.41) | 227 (37.15) |  |
| Fair | 698 (49.05) | 321 (52.54) |  |
| Poor | 150 (10.54) | 63 (10.31) |  |
| Smoking status, n (%) |  |  | 0.689 |
| Never | 1165 (81.87) | 508 (83.14) |  |
| Former | 67 (4.71) | 24 (3.93) |  |
| Current | 191 (13.42) | 79 (12.93) |  |
| Alcohol drinking, n (%) |  |  | 0.732 |
| Never | 1127 (79.20) | 491 (80.36) |  |
| Former | 61 (4.29) | 22 (3.60) |  |
| Current | 235 (16.51) | 98 (16.04) |  |
| Tooth brushing frequency, n (%) |  |  | 0.113 |
| ≤1 time/day | 582 (40.90) | 273 (44.68) |  |
| ≥2 times/day | 841 (59.10) | 338 (55.32) |  |
| Halitosis, n (%) |  |  |  |
| No | 994 (69.85) | 405 (66.28) | 0.111 |
| Yes | 429 (30.15) | 206 (33.72) |  |
| Butter tea consumption, n (%) |  |  | 0.551 |
| Never | 936 (65.78) | 417 (68.25) |  |
| Occasionally (<3 times/week) | 435 (30.57) | 174 (28.48) |  |
| Frequently (≥3 times/week) | 52 (3.65) | 20 (3.27) |  |
| Vegetable consumption, n (%) |  |  | 0.449 |
| Never | 18 (1.26) | 12 (1.96) |  |
| Occasionally (<3 times/week) | 512 (35.98) | 224 (36.66) |  |
| Frequently (≥3 times/week) | 893 (62.75) | 375 (61.37) |  |
| Previous Hp infection, n (%) |  |  | 0.463 |
| No | 1171 (82.29) | 511 (83.63) |  |
| Yes | 252 (17.71) | 100 (16.37) |  |
| Family history of Hp infection, n (%) |  |  | 0.701 |
| No | 1025 (72.03) | 435 (71.19) |  |
| Yes | 398 (27.97) | 176 (28.81) |  |
| History of peptic ulcer, n (%) |  |  | 0.159 |
| No | 1325 (93.11) | 558 (91.33) |  |
| Yes | 98 (6.89) | 53 (8.67) |  |
| History of chronic gastritis, n (%) |  |  | 0.564 |
| No | 1102 (77.44) | 321 (22.56) |  |
| Yes | 321 (22.56) | 145 (23.73) |  |
| Hp infection, n (%) |  |  |  |
| Negative | 674 (47.36) | 295 (48.28) | 0.704 |
| Positive | 749 (52.64) | 316 (51.72) |  |

Continuous variables are presented as median (interquartile range [IQR]) and categorical variables as number (percentage). Differences between groups were compared using the Mann–Whitney U test for continuous variables and the chi-square test or Fisher’s exact test for categorical variables. BMI, body mass index; Hp, Helicobacter pylori.

**Table S2. Univariable logistic regression analysis and multicollinearity assessment of predictors meeting the screening criterion for Hp infection**

| **Variable** | **OR (95% CI)** | **P value** | **VIF** |
| --- | --- | --- | --- |
| Age, years | 0.992 (0.986–0.998) | 0.007 | 2.771 |
| Weight change within 1 year |  |  |  |
| Stable (change <5 kg) | Reference | — |  |
| Gain >5 kg | 0.736 (0.517–1.048) | 0.089 | 1.081 |
| Loss >5 kg | 0.629 (0.398–0.994) | 0.047 | 1.049 |
| Ethnicity |  |  |  |
| Han | Reference | — |  |
| Hui | 1.055 (0.762–1.461) | 0.747 | 2.053 |
| Tibetan | 5.263 (3.912–7.080) | <0.001 | 4.634 |
| Other | 0.836 (0.481–1.454) | 0.526 | 1.080 |
| Monthly income  (per category increase) | 0.611 (0.534–0.700) | <0.001 | 2.044 |
| Frequency of out-of-province  travel within 1 year  (≥ 3 vs. ≤2 times/year) | 0.574 (0.484–0.680) | <0.001 | 1.483 |
| Educational level  (per category increase) | 0.625 (0.572–0.682) | <0.001 | 2.395 |
| Residence |  |  |  |
| Rural area | Reference | — |  |
| County town | 1.580 (1.171–2.132) | 0.003 | 1.785 |
| Urban | 0.902 (0.687–1.184) | 0.456 | 2.149 |
| Married status |  |  |  |
| Married | Reference | — |  |
| Unmarried/Divorced/Widowed | 1.270 (1.018–1.585) | 0.034 | 2.100 |
| Occupation |  |  |  |
| Government employee | Reference | — |  |
| Farmer | 2.477 (1.537–3.991) | <0.001 | 4.406 |
| Student | 1.845 (1.153–2.952) | 0.011 | 5.245 |
| Healthcare worker | 0.784 (0.478–1.284) | 0.333 | 2.866 |
| Worker | 1.523 (0.879–2.640) | 0.134 | 2.210 |
| Service worker | 0.911 (0.464–1.785) | 0.785 | 1.707 |
| Other | 1.613 (1.034–2.517) | 0.035 | 4.652 |
| Weekly working hours | 0.982 (0.977–0.987) | <0.001 | 1.672 |
| Work/family stress  (per category increase) | 0.766 (0.655–0.897) | 0.001 | 1.415 |
| Late-night sleep  (per category increase) | 0.819 (0.683–0.982) | 0.031 | 1.471 |
| Sleep quality  (per category increase) | 0.651 (0.553–0.766) | <0.001 | 1.340 |
| Drinking water source |  |  |  |
| Purified water | Reference | — |  |
| Tap water | 1.637 (1.320–2.030) | <0.001 | 1.283 |
| Well water | 3.253 (0.854–12.393) | 0.084 | 1.068 |
| Eating out frequency  (per category increase) | 0.773 (0.638–0.937) | 0.009 | 1.613 |
| Tea consumption  (per category increase) | 1.279 (1.095–1.494) | 0.002 | 1.278 |
| Butter tea consumption  (per category increase) | 2.065 (1.689–2.525) | <0.001 | 1.723 |
| Soy product consumption  (per category increase) | 0.627 (0.506–0.777) | <0.001 | 1.291 |
| Spicy food consumption  (per category increase) | 0.785 (0.647–0.952) | 0.014 | 1.391 |
| Raw food consumption  (per category increase) | 1.540 (1.255–1.891) | <0.001 | 1.345 |
| Cold food consumption  (per category increase) | 1.251 (1.015–1.542) | 0.036 | 1.387 |
| Fruit consumption  (per category increase) | 0.644 (0.527–0.788) | <0.001 | 1.492 |
| Vegetable consumption  (per category increase) | 0.495 (0.400–0.611) | <0.001 | 1.702 |
| Lamb consumption  (per category increase) | 0.584 (0.485–0.703) | <0.001 | 1.412 |
| Pork consumption  (per category increase) | 0.722 (0.630–0.829) | <0.001 | 2.194 |
| Tooth brushing frequency  (≥2 vs. ≤1 times/day) | 0.400 (0.322–0.498) | <0.001 | 1.239 |
| Hand hygiene before meals  and after toileting  (yes vs. no) | 0.350 (0.247–0.498) | <0.001 | 1.286 |
| Pet ownership  (yes vs. no) | 0.686 (0.501–0.940) | 0.019 | 1.057 |
| Use of serving chopsticks at home  (yes vs. no) | 1.318 (1.016–1.710) | 0.037 | 1.118 |
| Cleaning vegetables  before consumption  (yes vs. no) | 0.681 (0.492–0.941) | 0.020 | 1.114 |
| Halitosis  (yes vs. no) | 2.432 (1.918–3.084) | <0.001 | 1.129 |
| Family history of Hp infection  (yes vs. no) | 1.729 (1.365–2.191) | <0.001 | 1.127 |
| Chronic gastritis  (yes vs. no) | 2.196 (1.691–2.852) | <0.001 | 1.120 |
| Annual health examination  (yes vs. no) | 0.670 (0.542–0.826) | <0.001 | 1.309 |
| High-altitude residence  (yes vs. no) | 8.011 (5.604–11.453) | <0.001 | 4.904 |

Predictors with a univariable *P* value <0.05 were retained for subsequent feature selection. For categorical predictors with more than two levels, a predictor was retained if at least one non-reference category had a *P* value <0.05. VIFs were calculated among the predictors meeting this criterion. As no VIF exceeded 10, all 35 retained predictors were entered into the LASSO regression model. Variables expressed as “per category increase” were analyzed as ordinal variables. Reference categories are indicated where applicable. OR, odds ratio; CI, confidence interval; VIF, variance inflation factor.
